# Leprosy: General Conclusions

**Published:** 1898-02

**Authors:** 


### Leprosy: General Conclusions.

At the close of the debates of the international Leprosy Conference, Berlin, 1897, the Secretaries have the honour to present the following short report on the general conclusions of the Conference.

They believe that such a resume will be especially desirable to those members who have been delegated by their respective governments, and who have to make reports on the result of the Conference.

As might be expected, a considerable portion of the discussion has related to the bacillus Lepræ, which the Conference accepts as the Virus of Leprosy, and which for upwards of twenty-five years has been known to the scientific world through the important discovery of Hansen and the able investigations of Neisser.

The conditions under which the bacillus grows and develops are still unknown, as well as the way of its invasion into the human system; but from the discussion of the Conference, it seems probable that an unaminity of opinion will soon prevail in reference to its modes of subsequent dissemination within the human body.

Very interesting observations have been brought forward in connection with the elimination of the bacilli in large quantities by means of the skin and the nasal and buccal mucous membranes of lepers; it is desired that such observations be confirmed where opportunities occur.

The question is of a very great importance to those who are entrusted with the care of the public health, as leprosy is now acknowledged to be a contagious disease.

Every leper is a danger to his surroundings, the danger varying with the nature and extent of his relations therewith, and also with the sanitary conditions under which he lives.

Although among the lower classes, every leper is especially dangerous to his family of fellow workers, cases of leprosy frequently appear in the higher social circles.

The theory of heredity of leprosy is now further shown to have lost ground, in comparison with the at present generally accepted theory of its contagiousness.

The treatment of leprosy has only had palliative results up to the present time.

Serum therapy has so far been unsuccessful.

In view of the virtual incurability of leprosy and the serious and detrimental effects which its existence in a community causes, and considering the good results which have followed the adoption of legal measures of issolation in Norway, the Leprosy Conference, as a logical issue of the theory that the disease is contagious, has adopted the following resolution proposed by Dr. Hansen and amended by Dr. Besnier.

1. In such countries, where leprosy forms foci or has a great extension, we have in isolation the best means of preventing the spread of the disease.

2. The system of obligatory notification, of observation and isolation as carried out in Norway, is recommended to all nations with local self-government and a sufficient number of health officers.

3. It should be left to the legal authorities after consultation with the medical authorities to take such measures as are applicable to the special social conditions of the districts.

(Signed): SECRETARIES OF THE CONFERENCE:

PHIN. S. ABRAHAM, London. ED. ARNIG, Hamburg.
A. VON BERGMANN, Riga. J. J. KINYOUN, Washington.
E. DUBOIS HAVENITH, Bruxelles. G. THIBIERGE, Paris.
EDV. EHLERS, Copenhagen, General Secretary.

